# Supplementary material for: IL-27 Signaling Promotes Th1 Response by Downregulating IL-10 Production in DCs during Chlamydial Respiratory Infection
Source: Microorganisms. 2023 Feb 27;11(3):604. doi: 10.3390/microorganisms11030604 (PMC10051384; doi:10.3390/microorganisms11030604)
Supplement: Supplementary file 1 [file microorganisms-11-00604-s001.zip › microorganisms-2217484-supplementary.pdf]

IL-27 signaling promotes Th1 response by downregulating IL-10 production in DCs during chlamydial respiratory infection

Jiajia Zeng[1†], Shuaini Yang[1†], Yuqing Tuo[1], Xiaoyu Zha[1], Ruoyuan Sun[1], Tingsha Lu[1], Hong Zhang[1], Lu Tan[1], Sai Qiao[1], Jing Yang[2,*] and Hong Bai[1,*]

[1] Key Laboratory of Immune Microenvironment and Disease (Ministry of Education), Department of Immunology, School of Basic Medical Sciences, Tianjin Medical University, Tianjin 300070, China.

[2] Tianjin Key Laboratory of Acute Abdomen Disease Associated Organ Injury and ITCWM Repair, Institute of Acute Abdominal Diseases of Integrated Traditional Chinese and Western Medicine, Tianjin Nankai Hospital, Tianjin 300100, China.

[†] These authors have contributed equally to this work.

* Correspondence: Hong Bai (hongbai25@tmu.edu.cn) and Jing Yang (yj518629@126.com)

Supplementary Table S1. Sequences of primers used in this study.

Gene name	Forward primer (5'→3')	Reverse primer (5'→3')
β-actin	GGCTGTATTCCCCTCCATCG	CCAGTTGGTAACAATGCCATGT
p28	CTGTTGCTGCTACCCTTGCTT	CACTCCTGGCAATCGAGATTC
EBI3	TGAGGTTCAGGGCTATGTCC	GAAGTGTGGTAGCGAGGAAG
WSX-1	TCTGTCAGTTCCGGTACAAGG	GGTTCTGCATCTCAACAGGAGT
GP130	GCAAGTGTTCTCAAGGTCCGAGTC	GTGCCAAGAGCATCCGCTGTAG
IL-12p40	TGGTTTGCCATCGTTTTGCTG	ACAGGTGAGGTTCACTGTTTCT
IL-12p35	CAATCACGCTACCTCCTCTTTT	CAGCAGTGCAGGAATAATGTTTC
IL-10	CTTACTGACTGGCATGAGGATCA	GCAGCTCTAGGAGCATGTGG
IL-6	TGAACAACGATGATGCACTTGCAG	TAGCCACTCCTTCTGTGACTCCAG

Supplementary Table S2. Antibodies used for flow cytometry in this study.

Antibody	Fluor	Manufacturer
CD45	APC-Cy7	Biolegend
CD45	PerCP-Cy5.5	Biolegend
CD11c	APC	Biolegend
MHC II	PerCP-Cy5.5	Biolegend
CD40	PE-Cy7	Biolegend
CD80	PE	Biolegend
CD86	PE-Cy7	Biolegend
Ly6G	APC	Biolegend
CD4	APC	Biolegend
CD8	PerCP	Biolegend
IFN-γ	PE-Cy7	Biolegend
IL-17A	PE	Biolegend
IL-4	PE	Biolegend
CD11b	FITC	Biolegend
CD3	FITC	BD Biosciences

Supplementary Table S3. The scoring of semi-quantitative pathological.

Score	
0	Normal
1	Moderate and limited inflammatory granuloma with less than 25% cell infiltration and no obvious infiltration in adjacent alveolar septa or air space
2	Moderate interstitial pneumonia with 25%-50% cell infiltration and septal congestion and septal edema
3	Inflammatory cells infiltrated into perivascular, peribronchiolar, alveolar septa, and air space, accounting for 50%-75% of the area
4	Over 75% of the lung area infiltrated with inflammatory cells

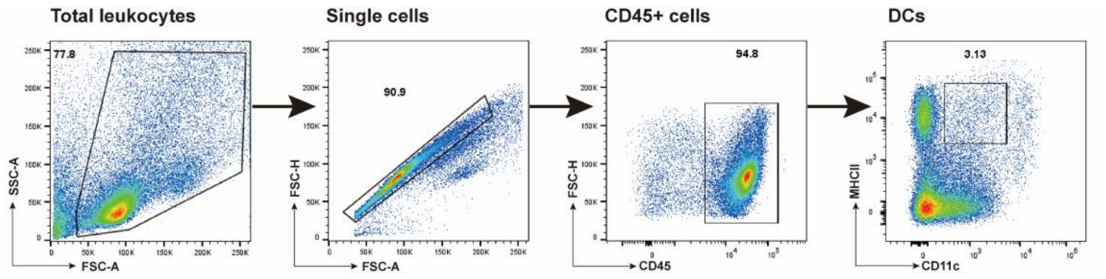

Supplementary Figure S1. The flow cytometry gate strategy for the analysis of dendritic cells (DCs). Related to Figure 1. An animal model of *C. muridarum* respiratory infection was induced by intranasally inoculating with 1×10^3 inclusion forming units (IFUs) *C. muridarum*. The lung and spleen single-cells of wild-type (WT) mice on days 0, 3, 7, and 14 post-infection (p.i.) were prepared. The pulmonary and splenic DCs were detected by flow cytometry, with DCs presented as $CD45^+CD11c^+MHCII^+$ cells.

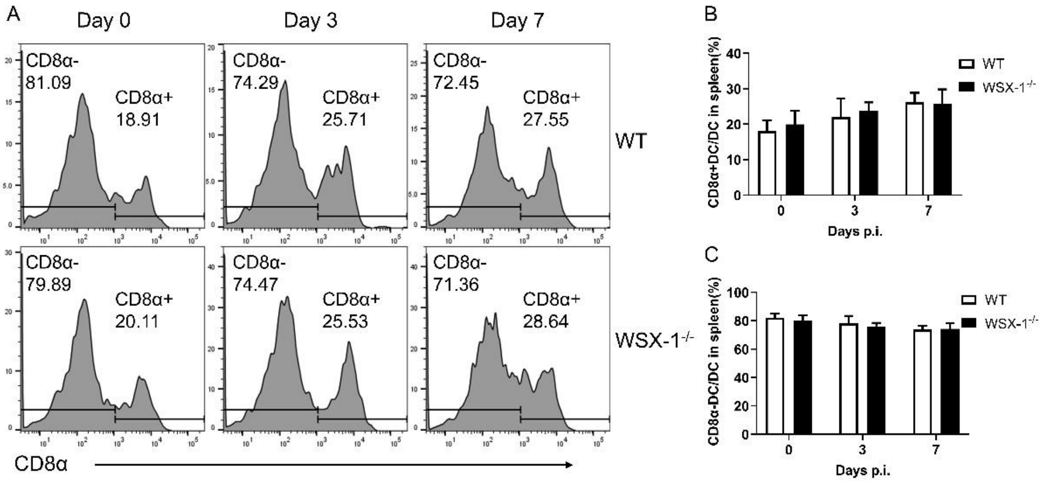

Supplementary Figure S2. Infiltrations of DC subsets in the spleen of WSX-1$^{-/-}$ mice following *Chlamydia muridarum (C. muridarum)* respiratory infection. An animal model of *C. muridarum* respiratory infection was induced by intranasally inoculating with 1×10^3 inclusion forming units (IFUs) *C. muridarum*. The spleen single cells of wild-type (WT) and WSX-1 deficient (WSX-1$^{-/-}$) mice on days 0, 3, and 7 post-infection (p.i.) were prepared. The frequencies of two splenic DC subsets were detected by flow cytometry, with cDC1 characterized as CD8α$^+$ cells from spleen DCs (CD45$^+$ CD11c$^+$ cells as described in Figure 1A), and cDC2 characterized as CD8α$^-$ cells. Representative flow cytometric images (A) and summaries of their percentages (B and C) were shown. Data are represented as means ± SD from n = 3 - 4 per group, representative of one of three independent experiments. Statistical significances of differences are determined by two-way ANOVA followed by Šidák's multiple comparisons test.

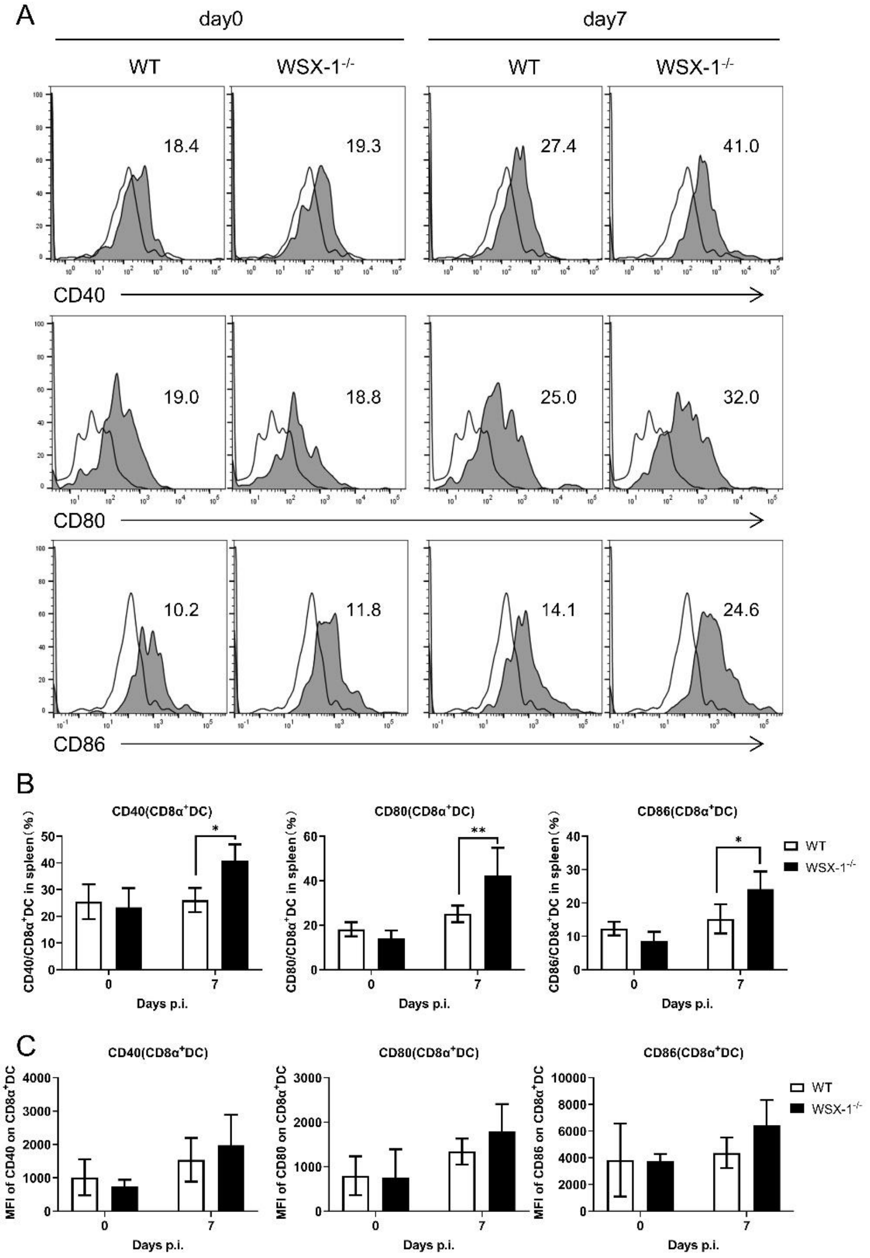

Supplementary Figure S3. Costimulatory molecules expression on CD8α$^+$ DCs in the spleen of WSX-1$^{-/-}$ mice following *C. muridarum* respiratory infection. The spleen single cells of WT and WSX-1$^{-/-}$ mice on days 0 and 7 p.i. were prepared. The

expression of costimulatory molecules CD40, CD80, and CD86 (shaded histogram) with fluorescence minus one (FMO) control (solid lines) on splenic CD8α$^+$ DC subset from WT and WSX-1$^{-/-}$ mice were analyzed by flow cytometry based on gated CD8α$^+$ DCs as described in Supplementary Figure 1(A). Representative flow cytometric images (A), summaries of their percentages (B), and mean fluorescence intensity (MFI) (C) were shown. Data are represented as means ± SD from n = 3 - 4 per group, representative of one of three independent experiments. Statistical significances of differences are determined by two-way ANOVA followed by Šidák's multiple comparisons test. *P < 0.05, **P < 0.01.

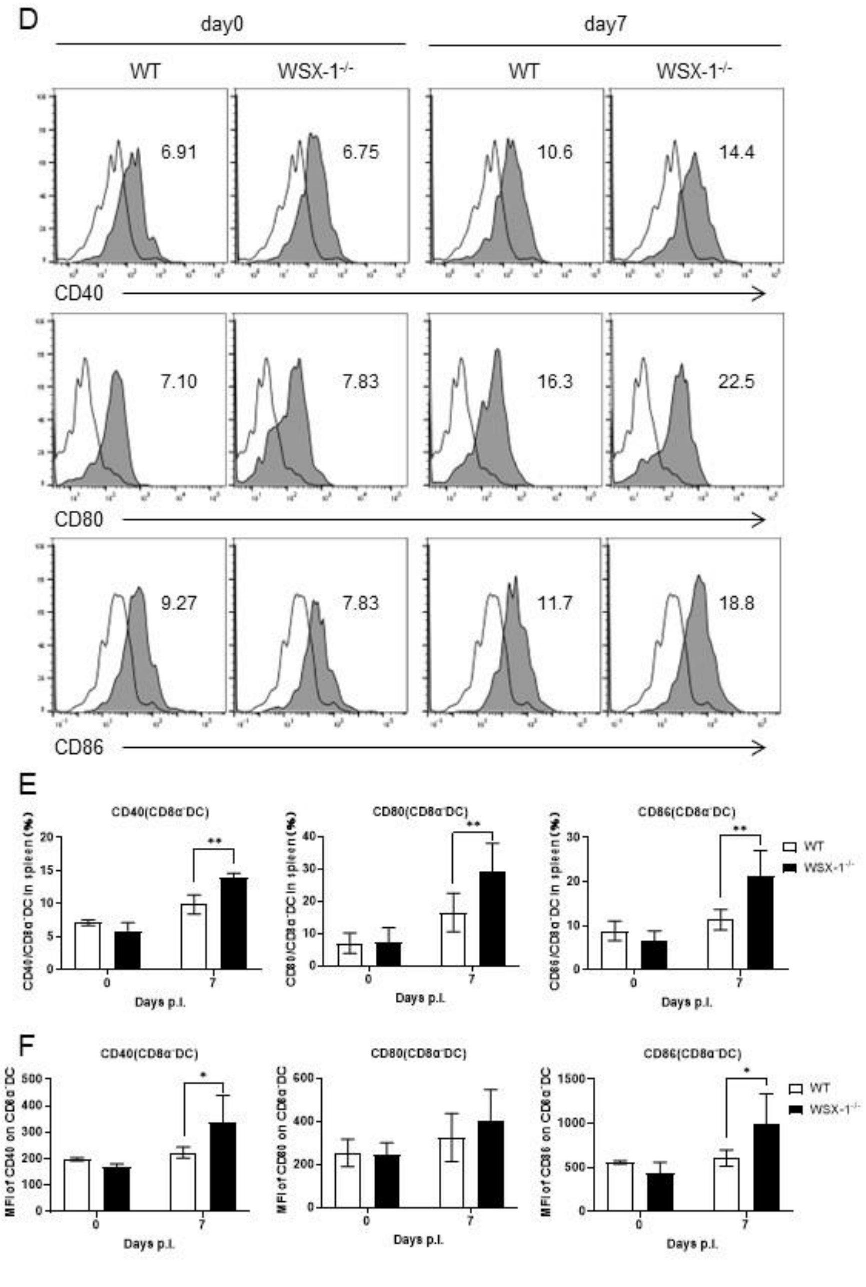

Supplementary Figure S4. Costimulatory molecules expression on CD8α^{-} DCs in the spleen of WSX-1$^{-/-}$ mice following *C. muridarum* respiratory infection. The spleen single cells of WT and WSX-1$^{-/-}$ mice on days 0 and 7 p.i. were prepared. The

expression of costimulatory molecules CD40, CD80, and CD86 (shaded histogram) with fluorescence minus one (FMO) control (solid lines) on splenic CD8α$^-$ DC subset from WT and WSX-1$^{-/-}$ mice were analyzed by flow cytometry based on gated CD8α$^-$ DCs as described in Supplementary Figure 1(A). Representative flow cytometric images (A), summaries of their percentages (B), and mean fluorescence intensity (MFI) (C) were shown. Data are represented as means ± SD from n = 3 - 4 per group, representative of one of three independent experiments. Statistical significances of differences are determined by two-way ANOVA followed by Šidák's multiple comparisons test. *P < 0.05, **P < 0.01.
